# Association Study of Single Nucleotide Polymorphisms in *XRCC1* Gene with Risk of Hepatocellular Carcinoma in Chinese Han Population

**DOI:** 10.1155/2013/138785

**Published:** 2013-07-30

**Authors:** Jingwang Bi, Chen Zhong, Kainan Li, Huili Chu, Baocheng Wang

**Affiliations:** Department of Oncology, General Hospital of Jinan Military Region, Jinan, Shangdong 250031, China

## Abstract

Hepatocellular carcinoma (HCC) is one of the most frequently causing cancer-related deaths worldwide. Previous evidence suggests that the X-ray repair cross-complementing group 1 gene (*XRCC1*) is an important candidate gene for influencing the risk of HCC. The aim of this study was to assess the association of *XRCC1* genetic polymorphisms with the risk of HCC in Chinese Han population. A total of 1314 subjects, including 651 HCC patients and 663 healthy controls, were enrolled in this case-control study. Two genetic variants (c.1254C>T and c.1517G>C) in *XRCC1* gene were genotyped by created restriction site-polymerase chain reaction (CRS-PCR) and PCR-restriction fragment length polymorphism (PCR-RFLP) methods. Our data indicated that the allele and genotype frequencies of these two genetic variants were statistical difference in HCC cases and healthy controls. Association analyses suggested that these two genetic variants were statistically associated with the increased risk of HCC in all genetic models (for c.1254C>T, TT versus CC: OR = 2.30, 95% CI 1.61–3.28; CT versus CC: OR = 1.32, 95% CI 1.05–1.67; TT/CT versus CC: OR = 1.50, 95% CI 1.20–1.86; TT versus CT/CC: OR = 2.00, 95% CI 1.43–2.80; T versus C: OR = 1.47, 95% CI 1.25–1.73; for c.1517G>C, CC versus GG: OR = 1.90, 95% CI 1.34–2.69; GC versus GG: OR = 1.56, 95% CI 1.24–1.97; CC/GC versus GG: OR = 1.63, 95% CI 1.31–2.03; CC versus GC/GG: OR = 1.52, 95% CI 1.10–2.11; C versus G: OR = 1.45, 95% CI 1.23–1.70). The allele-T of c.1254C>T and allele-C of c.1517G>C genetic variants may contribute to HCC susceptibility in Chinese Han population.

## 1. Introduction

Hepatocellular carcinoma (HCC) is the fifth common solid tumor and the third leading cause of cancer-related deaths in the world [1–3]. It is indicated that the estimated incidence of new HCC cases is approximately 500.000–1 000.000 [1]. It causes 600.000 deaths globally every year [1]. Emergence evidence indicates that HCC has significantly geographical variation, and China has a very high incidence with approximately 55% of annual new cases of HCC in the world [1, 4]. Since the 1990s, HCC has been one of the most common causes of cancer-related deaths in China [5, 6]. Many environmental factors, such as chronic hepatitis B (HBV) or hepatitis C (HCV) viral infections, alcohol consumption, cigarette smoking, and diabetes mellitus, have been evaluated the effects on HCC susceptibility [7–10], while genetic factors might play the key roles in the pathogenesis of HCC [11, 12]. To date, the exact mechanism of hepatocarcinogenesis still remains poorly understood. It has been reported that the human X-ray repair cross-complementing group 1 gene (*XRCC1*) is an important candidate gene influencing HCC susceptibility [13–25]. Several previous studies have been approved to evaluate the possible association between the single nucleotide polymorphisms (SNPs) in *XRCC1* gene (such as arginine (Arg)194 tryptophan (Trp), Arg280 histidine (His), and Arg399 glutamssine (Gln)) and the risk of HCC [13–20]. However, the association of the c.1254C>T and c.1517G>C SNPs in *XRCC1* gene with HCC susceptibility has not been investigated. Thus, in this case-control study, the objective of this study was to assess whether these two genetic variants influencing the susceptibility of HCC in Chinese Han population.

## 2. Materials and Methods

### 2.1. Subjects

A total of 651 cases with HCC at the General Hospital of Jinan Military Region were enrolled from January 2009 to August 2012 in this study. Subjects with HCC were diagnosed by doctors based on the standards established by Chinese Society of Liver Cancer (CSLC). The control group consisted of 663 health subjects selected from health screening program participants, which have been excluded those with medical history of surgery, cancer, and other diseases. All subjects were unrelated Chinese Han nationality. The clinical characteristics, including gender, age, alcohol drinking, tobacco smoking, hypertension, diabetes mellitus, family history of HCC, serum a-FP levels, and HBV serological markers, were collected in Table 1. This study was approved by the Institutional Ethics Committee of General Hospital of Jinan Military Region, and informed consent for participation from all subjects was obtained.

### 2.2. DNA Extraction and PCR Amplification

Genomic DNA was extracted from the peripheral venous blood samples using the standard method [26]. Based on the mRNA sequences (GenBank ID: NM_006297.2) and DNA sequences (GenBank ID: NC_000019.9) of human *XRCC1 *gene, the specific polymerase chain reaction (PCR) primers were designed by Primer Premier 5.0 software. Primers, annealing temperature, fragment size, and region are shown in Table 2. The PCR were performed in a total volume of 20 *μ*L solution containing 50 ng template DNA, 1× buffer (Tris-HCl 100 mmol/L, pH 8.3; KCl 500 mmol/L), 0.25 *μ*mol/L primers, 2.0 mmol/L MgCl_2_, 0.25 mmol/L dNTPs, and 0.5 U Taq DNA polymerase (Promega, Madison, WI, USA). The PCR conditions consisted of 94°C for 5 min, followed by 35 cycles of 94°C for 35 seconds, annealing at the corresponding temperature (shown in Table 2) for 35 seconds and 72°C for 35 seconds, and a final extension at 72°C for 5 minutes. The PCR amplified products were separated by 2.0% agarose gel electrophoresis after ethidium bromide staining and then observed under UV light.

### 2.3. *XRCC1 *Genotyping

The genotyping for c.1254C>T SNP was detected by created restriction site-PCR (CRS-PCR) method with one of the primers containing a nucleotide mismatch, which enables the use of restriction enzymes for discriminating sequence variations [27–31]. The genotyping for c.1517G>C SNP was investigated by PCR-restriction fragment length polymorphism (PCR-RFLP) method. According to the supplier's manual, aliquots of 5 *μ*L PCR amplified products were digested with 2 U selected restriction enzyme (MBI Fermentas, St. Leon-Rot, Germany, shown in Table 2) at 37°C for 10 hours. The digested products were separated by electrophoresis in 2.5% agarose gel after ethidium bromide staining and observed under UV light for analyzing the *XRCC1* SNPs genotyping. DNA sequencing method was done at TaKaRa Biotechnology Co., Ltd. (Dalian, China) for almost 10% of the random samples showing *XRCC1 *genetic polymorphisms to ensure the concordance of the results from CRS-PCR and PCR-RFLP methods.

### 2.4. Statistical Analysis

All statistical analyses were analyzed by the Statistical Package for Social Sciences software (SPSS, Windows version release 15.0; SPSS Inc.; Chicago, IL, USA). The chi-squared (*χ* ^2^) test was used to assess the Hardy-Weinberg equilibrium in allele and genotype frequencies and general characteristics between case and control groups. The odds ratios (ORs) and 95% confidence intervals (95% CIs) from multivariate logistic regression were utilized to assess the strength of associations between genetic variants of *XRCC1* gene and the risk of HCC. A two-tailed significance level of P less than 0.05 was defined as statistically significant for all statistical tests.

## 3. Results

### 3.1. General Characteristics of the Subjects

In this case-control study, a total of 1314 subjects were enrolled consisted of 651 HCC cases and 663 healthy controls. The results from *χ* ^2^ test suggested that there are no significant differences between HCC cases and healthy controls with terms of age and gender distribution. As for other general characteristics of the subjects, such as smoking status and drinking consumption, diabetes mellitus, and hypertension, no significant differences were found between HCC cases and healthy controls. The general characteristics of the subjects are performed in Table 1.

### 3.2. Genotyping of *XRCC1 *SNPs

In the current study, two genetic variants (c.1254C>T and c.1517G>C) could be genotyped by CRS-PCR and PCR-RFLP methods, respectively, and confirmed by DNA sequencing. According to the DNA sequence analysis, the c.1254C>T genetic variant is a synonymous mutation, which caused by C to T mutations in exon 11 of human *XRCC1* gene (p-serine (Ser) 418Ser). As for c.1517G>C, this genetic variant is nonsynonymous mutation, which caused by G to C mutations in exon 14 of human *XRCC1* gene and led to the glycine (Gly) to alanine (Ala) amino acid replacement (p.Gly506Ala, Reference sequences GenBank IDs: NC_000019.9, NM_006297.2, and NP_006288.2). The PCR amplified products of c.1254C>T were digested with *Hpa*II restriction enzyme and divided into three genotypes, CC (195 and 23 bp), CT (218, 195, and 23 bp), and TT (218 bp). The PCR amplified products of c.1517G>C were digested with *Hae*III restriction enzyme and divided into three genotypes, GG (247 bp), GA (247, 168, and 79 bp), and AA (168, and 79 bp, Table 2).

### 3.3. Allele and Genotype Frequencies

The observed genotype frequencies of c.1254C>T and c.1517G>C genetic variants sites were corresponded to Hardy-Weinberg equilibrium in both of HCC cases and controls (*P* > 0.05, Table 3). Table 3 shows the allelic and genotypic frequencies of these two genetic variants. The C-allele of c.1254C>T and G-allele of c.1517G>C genetic variants were predominant alleles in the studied subjects. As for c.1254C>T, significantly differences were detected between the allele frequencies of HCC cases (C, 61.06%; T, 38.94%) and those of the healthy controls (C, 69.76%; T, 30.24%, *χ* ^2^ = 21.9827, *P* < 0.001), and the genotypic frequencies in HCC cases were statistically significant different from those of healthy controls (*χ* ^2^ = 22.3180, *P* < 0.001, Table 3). Similarly, as for c.1517G>C, significantly differences were found between the allele frequencies of HCC cases (G, 61.29%; C, 38.71%) and those of the healthy controls (G, 69.61%; C, 30.39%, *χ* ^2^ = 20.1085, *P* < 0.001), and the genotypic frequencies in HCC cases were statistically significant different from those of healthy controls (*χ* ^2^ = 20.3680, *P* < 0.001, Table 3).

### 3.4. Association between *XRCC1* SNPs and HCC Risk

Table 4 presents the association between *XRCC1* SNPs and the risk of HCC. As for c.1254C>T, there were statistically increased risk of HCC in the homozygote comparison (TT versus CC: OR = 2.30, 95% CI 1.61–3.28, *χ* ^2^ = 21.59, *P* < 0.001), heterozygote comparison (CT versus CC: OR = 1.32, 95% CI 1.05–1.67, *χ* ^2^ = 5.58, *P* = 0.018), dominant model (TT/CT versus CC: OR = 1.50, 95% CI 1.20–1.86, *χ* ^2^ = 12.96, *P* < 0.001), recessive model (TT versus CT/CC: OR = 2.00, 95% CI 1.43–2.80, *χ* ^2^ = 16.73, *P* < 0.001), and allele comparison (T versus C: OR = 1.47, 95% CI 1.25–1.73, *χ* ^2^ = 21.97, *P* < 0.001). As for c.1517G>C, significantly increased risk of HCC was found in the homozygote comparison (CC versus GG: OR = 1.90, 95% CI 1.34–2.69, *χ* ^2^ = 13.22, *P* < 0.001), heterozygote comparison (GC versus GG: OR = 1.56, 95% CI 1.24–1.97, *χ* ^2^ = 14.04, *P* < 0.001), dominant model (CC/GC versus GG: OR = 1.63, 95% CI 1.31–2.03, *χ* ^2^ = 19.15, *P* < 0.001), recessive model (CC versus GC/GG: OR = 1.52, 95% CI 1.10–2.11, *χ* ^2^ = 6.33, *P* = 0.012), and allele comparison (C versus G: OR = 1.45, 95% CI 1.23–1.70, *χ* ^2^ = 20.10, *P* < 0.001).

## 4. Discussion

It is general accepted that HCC is a common primary malignant liver cancer causing by complex interactions between genetic and environmental factors [7, 32–34]. There were several studies indicated that the genotypic variation plays key roles in human phenotypic variability for HCC susceptibility. The *XRCC1* gene is one of the most important candidate genes for HCC, and the association between several SNPs in *XRCC1* gene such as Arg194Trp, Arg280His and Arg399Gln and the risk of HCC have been assessed in recent years [13–20]; however, the results from these observations still remain conflicting rather than conclusive. Kiran et al. demonstrated that Arg194Trp, Arg280His, and Arg399Gln SNPs were significantly associated with the risk of hepatitis virus-related HCC in Indian population [16]. Pan et al. reported that the Arg399Cln Arg/Gln indicated an increased risk of HCC, especially for patients above 50 years old or with drinking habits in Chinese population [17]. Liu et al. indicated that the Arg399Gln is not associated with altered HCC susceptibility [21]. In the present study, on the basis of analysis of 651 HCC patients and 663 healthy control subjects, we detected the c.1254C>T and c.1517G>C SNPs of *XRCC1* gene by PCR-RFLP, CRS-PCR, and DNA sequencing methods. We demonstrated that these two SNPs have statistically significant impacts on the susceptibility to HCC in Chinese Han population (Table 4). Our data suggested that the significant difference were shown in the allelic and genotypic frequencies between HCC patients and healthy controls for these two SNPs (Table 3). As for c.1254C>T, the TT genotype was statistically associated with the increased susceptibility of developing HCC compared to CC genotype and CT/CC carriers (all *P* < 0.001). As for c.1517G>C, the CC genotype was statistically associated with the increased susceptibility of developing HCC compared to GG genotype and GC/GG carriers (all *P* < 0.001). Therefore, the T-allele of c.1254C>T and the C-allele of c.1517G>C genetic variants may contribute to the susceptibility of HCC (*P* < 0.001, Table 4). To the best of our knowledge, this is the first report regarding the association between c.1254C>T and c.1517G>C SNPs and the risk of HCC. Our data indicated that these two genetic variants were significantly associated with the increased susceptibility of HCC in Chinese Han population. The findings could provide new evidence for further analysis of the biological function role of *XRCC1* gene variants on the susceptibility of HCC carcinogenesis. Larger prospective investigations will be essential to confirm these results on different populations and explain the underlying molecular mechanism.

## Figures and Tables

**Table 1:** Clinical characteristics of hepatocellular carcinoma (HCC) cases and controls.

Characteristics	Cases (*n*)	%	Controls (*n*)	%	*χ* ^2^ value	*P* value
Number	651		663			
Gender (*n*)					0.6900	0.4062
Male	389	59.75	411	61.99		
Female	262	40.25	252	38.01		
Age (years)						
Mean ± SD	58.69 ± 12.45		59.17 ± 13.22		2.8181	0.0932
<55	365	56.07	402	60.63		
≥55	286	43.93	261	39.37		
Alcohol drinking					2.3325	0.1267
Yes	332	51.00	366	55.20		
No	319	49.00	297	44.80		
Tobacco smoking					0.8282	0.3628
Yes	349	53.61	372	56.11		
No	302	46.39	291	43.89		
Hypertension (*n*)					0.1742	0.6764
Yes	115	17.67	123	18.55		
No	536	82.33	540	81.45		
Diabetes mellitus (*n*)					1.0300	0.3102
Yes	121	18.59	138	20.81		
No	530	81.41	525	79.19		
Family history of HCC (*n*)						
Yes	48	7.37	—			
No	603	92.63	—			
Serum a-FP levels						
<400 ng/mL	206	31.64	—			
>400 ng/mL	445	68.36	—			
HBV serological markers (*n*)						
HBs Ag (+)	131	20.12	—			
HBs Ag (−)	520	79.88	—			

**Table 2:** Primers, PCR-RFLP, and CRS-PCR analyses for genotyping *XRCC1 *gene polymorphisms.

SNPs	Primer sequences	Annealing temperature (°C)	Amplification fragment (bp)	Region	Restriction enzyme	Genotype (bp)
c.1254C>T	5′-GAGGAGGATGAGGCCTCTCACAC-3′ 5′-TAAGGAGGGAGAGTGGGTGGGT-3′	63.9	218	Exon11	*Hpa*II	CC: 195, 23 CT: 218, 195, 23 TT: 218

c.1517G>C	5′-CAAGTCCCAGCTGAGAACTGAG-3′ 5′-GCTGCTCTGCATGCTCACTC-3′	59.0	247	Exon14	*Hae*III	GG: 247 GC: 247, 168, 79 CC: 168, 79

Note: PCR means polymerase chain reaction; PCR-RFLP means PCR-restriction fragment length polymorphism; CRS-PCR means created restriction site PCR; SNPs mean single nucleotide polymorphisms. Underlined nucleotides mark nucleotide mismatches enabling the use of the selected restriction enzymes for discriminating sequence variations.

**Table 3:** The genotype and allele frequencies of *XRCC1* gene polymorphisms in cases and controls.

Groups	c.1254C>T							c.1517G>C						
	Genotype frequencies (%)			Allele frequencies (%)				Genotype frequencies (%)			Allele frequencies (%)			
	CC	CT	TT	C	T	*χ* ^2^	*P*	GG	GC	CC	G	C	*χ* ^2^	*P*
Cases (*n* = 651)	252 (38.71)	291 (44.70)	108 (16.59)	795 (61.06)	507 (38.94)	2.3434	0.3098	246 (37.79)	306 (47.00)	99 (15.21)	798 (61.29)	504 (38.71)	0.0575	0.9717
Controls (*n* = 663)	322 (48.57)	281 (42.38)	60 (9.05)	925 (69.76)	401 (30.24)	0.0136	0.9932	330 (49.77)	263 (39.67)	70 (10.56)	923 (69.61)	403 (30.39)	2.5860	0.2744

Total (*n* = 1314)	574 (43.68)	572 (43.53)	168 (12.79)	1720 (65.45)	908 (34.55)	1.8464	0.3972	576 (43.84)	569 (43.30)	169 (12.86)	1721 (65.49)	907 (34.51)	2.3218	0.3132
	*χ* ^2^ = 22.3180, *P* < 0.001			*χ* ^2^ = 21.9827, *P* < 0.001				*χ* ^2^ = 20.3680, *P* < 0.001			*χ* ^2^ = 20.1085, *P* < 0.001			

**Table 4:** Association between hepatocellular carcinoma (HCC) risk and *XRCC1 *gene polymorphisms.

SNPs	Comparisons	Test of association		
		OR (95% CI)	*χ* ^2^ value	*P* value
c.1254C>T	Homozygote comparison (TT vs. CC)	2.30 (1.61–3.28)	21.59	<0.001
	Heterozygote comparison (CT vs. CC)	1.32 (1.05–1.67)	5.58	0.018
	Dominant model (TT/CT vs. CC)	1.50 (1.20–1.86)	12.96	<0.001
	Recessive model (TT vs. CT/CC)	2.00 (1.43–2.80)	16.73	<0.001
	Allele contrast (T vs. C)	1.47 (1.25–1.73)	21.97	<0.001

c.1517G>C	Homozygote comparison (CC vs. GG)	1.90 (1.34–2.69)	13.22	<0.001
	Heterozygote comparison (GC vs. GG)	1.56 (1.24–1.97)	14.04	<0.001
	Dominant model (CC/GC vs. GG)	1.63 (1.31–2.03)	19.15	<0.001
	Recessive model (CC vs. GC/GG)	1.52 (1.10–2.11)	6.33	0.012
	Allele contrast (C vs. G)	1.45 (1.23–1.70)	20.10	<0.001

SNPs: single nucleotide polymorphisms; OR: odds ratio; CI: confidence interval; vs.: versus.
